# Acetic Acid Can Catalyze Succinimide Formation from Aspartic Acid Residues by a Concerted Bond Reorganization Mechanism: A Computational Study

**DOI:** 10.3390/ijms16011613

**Published:** 2015-01-12

**Authors:** Ohgi Takahashi, Ryota Kirikoshi, Noriyoshi Manabe

**Affiliations:** Faculty of Pharmaceutical Sciences, Tohoku Pharmaceutical University, 4-4-1 Komatsushima, Aoba-ku, Sendai 981-8558, Japan; E-Mails: kirikoshi@tohoku-pharm.ac.jp (R.K.); manabe@tohoku-pharm.ac.jp (N.M.)

**Keywords:** aspartic acid residue, nonenzymatic reaction, succinimide, density functional theory, computational chemistry, acetic acid, buffer catalysis, double proton transfer, concerted bond reorganization, protein drugs

## Abstract

Succinimide formation from aspartic acid (Asp) residues is a concern in the formulation of protein drugs. Based on density functional theory calculations using Ace-Asp-Nme (Ace = acetyl, Nme = NHMe) as a model compound, we propose the possibility that acetic acid (AA), which is often used in protein drug formulation for mildly acidic buffer solutions, catalyzes the succinimide formation from Asp residues by acting as a proton-transfer mediator. The proposed mechanism comprises two steps: cyclization (intramolecular addition) to form a *gem*-diol tetrahedral intermediate and dehydration of the intermediate. Both steps are catalyzed by an AA molecule, and the first step was predicted to be rate-determining. The cyclization results from a bond formation between the amide nitrogen on the *C*-terminal side and the side-chain carboxyl carbon, which is part of an extensive bond reorganization (formation and breaking of single bonds and the interchange of single and double bonds) occurring concertedly in a cyclic structure formed by the amide NH bond, the AA molecule and the side-chain C=O group and involving a double proton transfer. The second step also involves an AA-mediated bond reorganization. Carboxylic acids other than AA are also expected to catalyze the succinimide formation by a similar mechanism.

## 1. Introduction

**Scheme 1:**
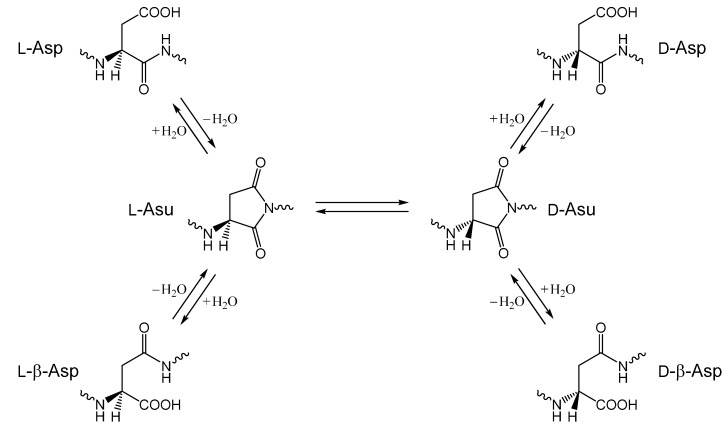
Nonenzymatic reactions of aspartic acid (Asp) residues via the succinimide intermediate (aminosuccinyl (Asu) residue).

Among nonenzymatic post-translational modifications of proteins, the alterations of normal l-aspartic acid (l-Asp) residues to l-β-Asp, d-Asp and d-β-Asp residues have recently attracted considerable attention because of relevance to aging and pathologies (especially those of age-related diseases, such as cataract and Alzheimer’s disease) [1,2,3,4,5,6,7,8,9,10,11,12]. These altered Asp residues are formed through a five-membered cyclic succinimide intermediate having an aminosuccinyl (Asu) residue instead of Asp (Scheme 1) [13,14]. The l-Asu intermediate, having two carbonyl groups in the succinimide moiety, can be hydrolyzed either back to l-Asp or to l-β-Asp, the structural isomer of l-Asp. Moreover, because the succinimide is racemization-prone [15,16,17], d-Asp and d-β-Asp residues can also be formed via d-Asu.

**Scheme 2:**
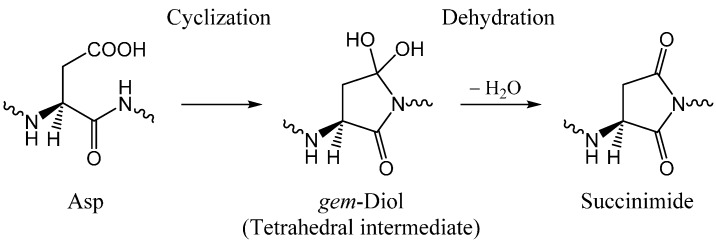
Two-step mechanism for succinimide formation from an Asp residue.

Succinimide formation has been regarded as a nucleophilic substitution at the carbonyl carbon comprised of two steps (an addition-elimination or a cyclization-dehydration mechanism) (Scheme 2) [18,19,20]. The first step is an intramolecular addition (cyclization) in which the amide nitrogen of the *C*-terminal peptide bond nucleophilically attacks the carboxyl carbon of the Asp side chain. This gives a tetrahedral intermediate, which is probably a *gem*-diol at neutral to acidic pH [20,21]. In the second step, a water molecule is eliminated from the *gem*-diol group. Both steps are thought to require a catalyst, because density-functional quantum-chemical calculations show that the energy barriers are too high without a catalyst [20,21]. Water is a good candidate as a catalyst of the succinimide-forming reactions, as we have recently shown computationally [22,23,24].

Buffers may also catalyze the succinimide-forming reactions. When hen egg-white lysozyme was incubated at pH 4.0 (acetate buffer) and at 40 °C, conversion of the Asp101 residue to the Asu form was observed [25]. The formation of the Asu residue increased and saturated as the concentration of acetate buffer was increased. This may indicate that acetic acid (AA) or acetate ion acts as a catalyst in succinimide formation. It should be noted that Asp101 is located in a structurally flexible region on the surface of the lysozyme molecule; in particular, its side chain is conformationally disordered and also the carboxyl group is highly solvent-accessible [26]. It is also notable that the Asu-containing lysozyme can be isolated as crystals [27,28].

From a mechanistic point of view, there is another concern. Amide nitrogens are thought to be poor nucleophiles, because of the amide resonance (conjugation with the electron-withdrawing carbonyl group) [29]. Indeed, nucleophilic attacks by amide nitrogens are not very common in synthetic organic chemistry [30,31,32,33,34]. Can the nucleophilic attacks by amide nitrogens really occur under mild conditions? Recently, we have computationally shown the possibility that cyclization to give a *gem*-diol tetrahedral intermediate occurs after the amide group is converted to the tautomeric iminol form [22,23,24]. The π electron density of the nitrogen atom is thought to be increased by iminolization, enhancing its nucleophilicity.

The alterations of l-Asp residues also occur in protein drugs, such as monoclonal antibodies [35,36,37,38,39,40,41,42,43,44,45,46,47,48,49], which may affect their stability, potency and/or safety, presenting challenges to the pharmaceutical industry during the process of protein purification, formulation, storage and delivery. Mildly acidic buffers have been widely used for the formulation of proteins, because many physical and chemical changes tend to be minimized at pH 4–5 [50,51]. However, it has been noticed that the succinimide intermediate is more stable (or its formation is faster) at mildly acidic pH than at higher pH [18,41,42,45,47,52,53,54]. Moreover, since AA is commonly used for mildly acidic buffers, the above-mentioned possibility of AA-catalyzed succinimide formation from Asp residues could be a pharmaceutical concern. On the other hand, α-aminosuccinimides themselves have received considerable attention in drug design [55].

**Figure 1:**
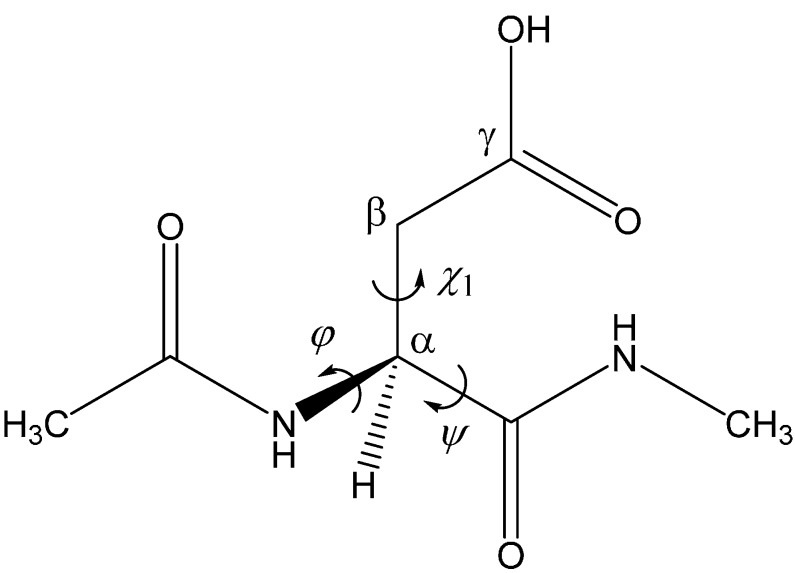
The model compound used in the present study (Ace-Asp-Nme). The φ (C–N–C_α_–C) and ψ (N–C_α_–C–N) dihedral angles, which characterize the main-chain conformation, and the χ_1_ dihedral angle (N–C_α_–C_β_–C_γ_), which characterizes the side-chain conformation, are indicated.

In this paper, we computationally show that an AA molecule (CH_3_COOH, not CH_3_COO^−^) can catalyze the two-step formation of succinimide from l-Asp residues (Scheme 2). As in our previous studies [22,23,24], Ace-Asp-Nme (Ace = acetyl, Nme = NHMe) (Figure 1) was employed as a model compound. Note that the Asp side chain is in the protonated form (–COOH), because only this form is thought to undergo nucleophilic attack by the backbone nitrogen atom to form the five-membered ring [14,18,47,49]. Although Asp residues exist essentially in the deprotonated form (–COO^−^) at neutral or physiological pH, the amount of the protonated form increases as pH decreases.

## 2. Results and Discussion

**Figure 2:**
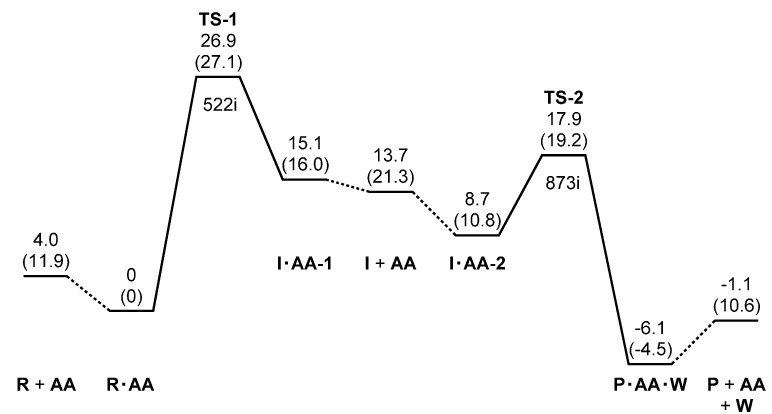
Energy diagram (kcal·mol^−1^), where relative energies corrected for the zero-point energy (ZPE) and the SM8 (solvation model 8) hydration free energy are shown with respected to the reactant complex, R•AA (R, reactant molecule; AA, acetic acid). The ZPE-corrected relative energies in a vacuum are shown in parentheses for comparison. The single imaginary frequency (cm^−1^) is also shown for TS-1 and TS-2 (TS, transition state). I, intermediate; P, product molecule; W, water.

**Figure 3:**
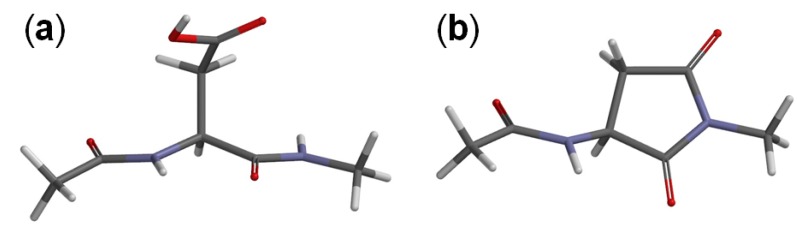
The geometries of (**a**) the reactant R (model compound, Figure 1) (φ = −162°, ψ = 162°, χ_1_ = 72°) and (**b**) the succinimide product P (φ = −171°, ψ = −141°, χ_1_ = 136°).

**Figure 4:**
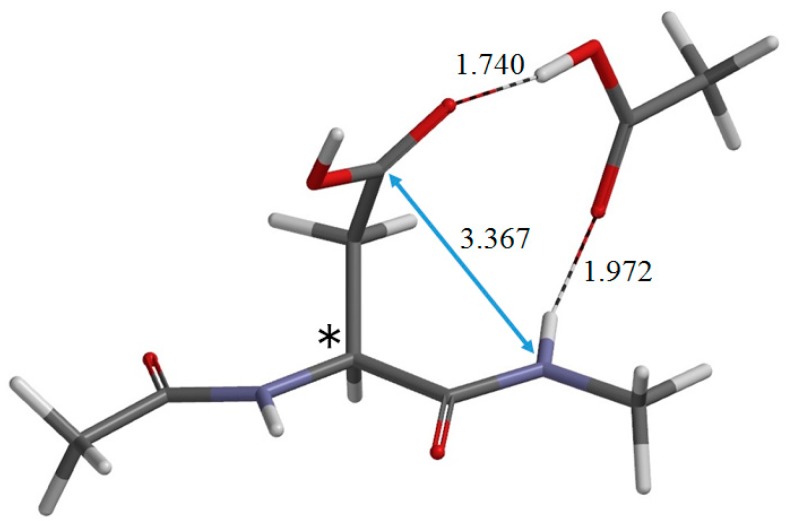
The geometry of the reactant complex R•AA (φ = −164°, ψ = −178°, χ_1_ = 76°). The α carbon atom is indicated by an asterisk (*). Selected interatomic distances are shown in Å. The gas-phase total energy of this geometry is −913.600369 *E*_h_.

**Figure 5:**
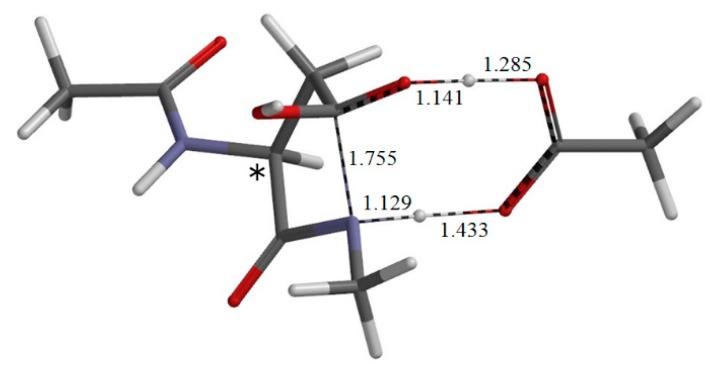
The geometry of the transition state TS-1 of the first step (cyclization) (φ = −162°, ψ = −150°, χ_1_ = 115°). The distances of forming and breaking bonds are shown in Å. The asterisk (*) indicates the α carbon.

**Figure 6:**
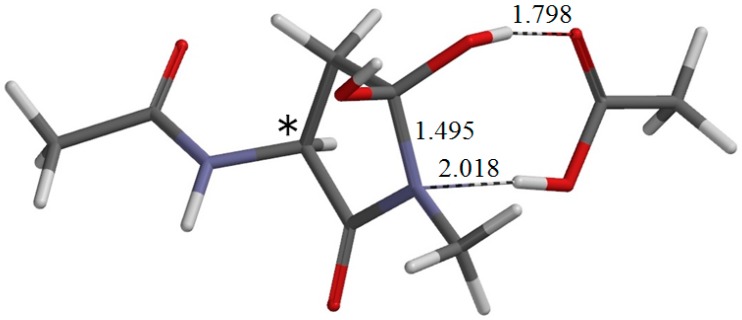
The geometry of I•AA-1 (φ = −167°, ψ = −138°, χ_1_ = 117°), which is the intermediate complex directly connected to TS-1. Selected interatomic distances are shown in Å. The asterisk (*) indicates the α carbon.

**Figure 7:**
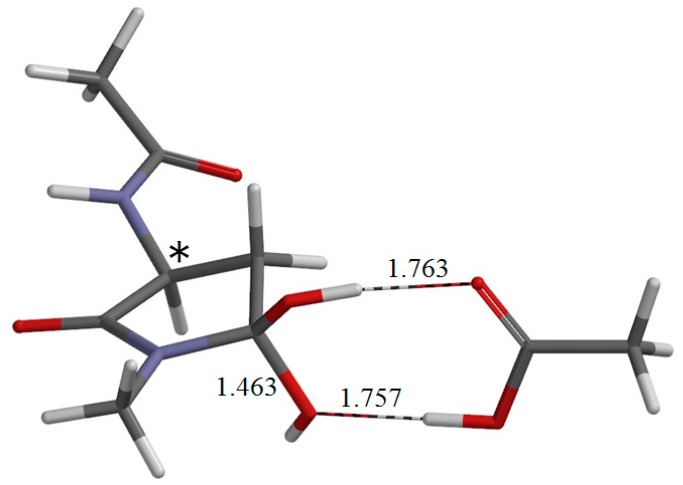
The geometry of I•AA-2 (φ = −170°, ψ = −145°, χ_1_ = 146°), which is the intermediate complex directly connected to TS-2. Selected interatomic distances are shown in Å. The asterisk (*) indicates the α carbon.

**Figure 8:**
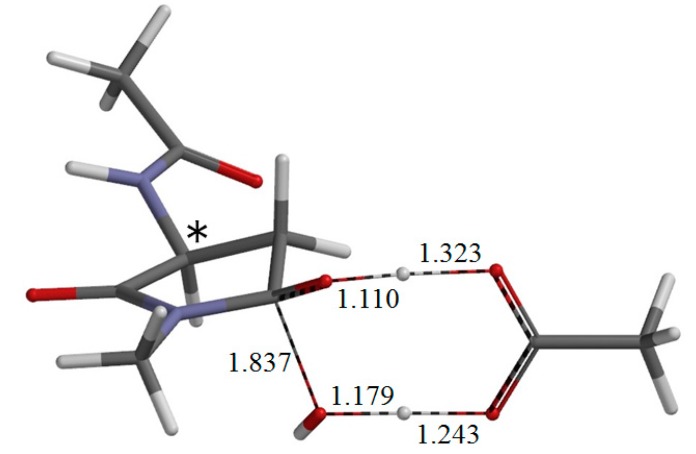
The geometry of the transition state TS-2 of the second step (dehydration) (φ = −169°, ψ = −142°, χ_1_ = 144°). The distances of forming and breaking bonds are shown in Å. The asterisk (*) indicates the α carbon.

**Figure 9:**
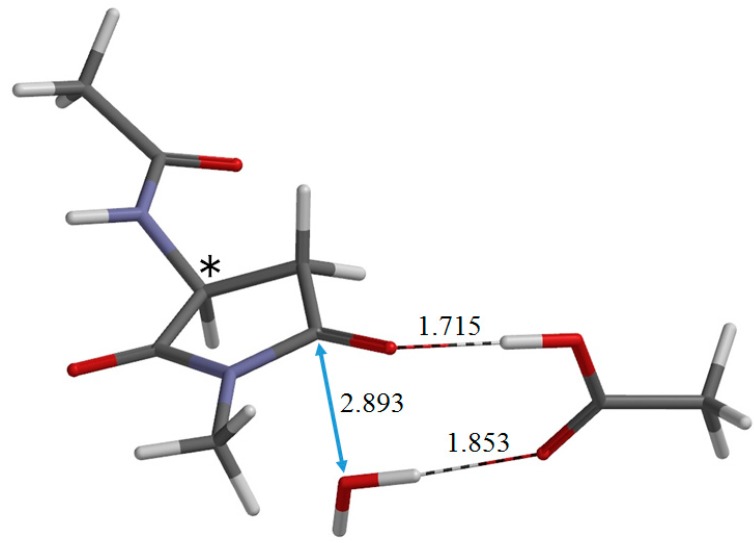
The geometry of the product complex P•AA•W (φ = −173°, ψ = −140°, χ_1_ = 137°) formed by the succinimide product P, the regenerated acetic acid molecule AA and the released water molecule W. Selected interatomic distances are shown in Å. The asterisk (*) indicates the α carbon.

Figure 2 shows the energy diagram for the two-step succinimide formation catalyzed by an AA molecule, and Figure 3, Figure 4, Figure 5, Figure 6, Figure 7, Figure 8 and Figure 9 show optimized geometries. The values of dihedral angles φ, ψ and χ_1_ (Figure 1) are shown in the captions to Figure 3, Figure 4, Figure 5, Figure 6, Figure 7, Figure 8 and Figure 9. R, I and P stand for the reactant (model compound), the *gem*-diol tetrahedral intermediate and the succinimide product, respectively. AA and W are acetic acid and water molecules, respectively. TS-1 and TS-2 are the transition states of the first and second steps (cyclization and dehydration), respectively. While geometry optimizations and zero-point energy (ZPE) calculations were performed in a vacuum, hydration free energies estimated by the SM8 (solvation model 8) continuum model [56,57] were taken into account in relative energy calculations. As may be seen from Figure 2, the effects of hydration on relative energies are small, except for complexation energies. The relative energies cited in the following are those in water, unless otherwise noted.

The reactant molecule R shown in Figure 3a is in an extended conformation with the backbone dihedral angles φ and ψ (Figure 1) being −162° and 162°, respectively. Note that this is not the most stable conformer, but the “reactive” conformer, in that it can form the reactant complex R•AA (a 1:1 complex between R and AA) (Figure 4), from which cyclization to the five-membered ring occurs. In R•AA, the AA molecule forms two hydrogen bonds to R, bridging the NH of the *C*-terminal peptide bond and the C=O of the side-chain carboxyl group. As a result, the distance between the NH nitrogen and the carboxyl carbon is 3.367 Å. Upon complexation between R and AA, the dihedral angle ψ changes by 20°, while changes in φ and χ_1_ are much smaller. The complexation energies calculated in a vacuum and in water are 11.9 and 4.0 kcal·mol^−1^, respectively.

TS-1 (Figure 5) is the transition state for cyclization from the reactant complex R•AA, and I•AA-1 (Figure 6) is the intermediate complex directly connected to TS-1. The distance of the forming C−N bond in TS-1 is 1.755 Å, and that of the newly formed C−N bond in I•AA-1 is 1.495 Å. Because of the intramolecular cyclization, changes in ψ and χ_1_ by about 40° occur continuously from R•AA to I•AA-1 through TS-1.

Concomitantly with the C–N bond formation, a double proton transfer mediated by the AA molecule occurs, so that the resultant intermediate species is a *gem*-diol having two OH groups on the C_γ_ atom. More specifically, the NH hydrogen moves toward the C=O oxygen of AA, the OH hydrogen of AA moves toward the C=O oxygen of the side chain and the single and double bonds are interchanged in the COO moiety of AA. The AA molecule thus acts as both proton donor and acceptor in the double proton transfer. Moreover, this double proton transfer is somewhat asynchronous; namely, the proton transfer from the AA molecule precedes that from the NH group. In the resultant complex, I•AA-1, there are two hydrogen bonds between I (the intermediate in its isolated state; geometry not shown) and AA. One is between the amide nitrogen in the five-membered ring and the OH of the newly-formed AA. The other is between the C=O of AA and the newly-formed OH in the *gem*-diol group.

The energy of TS-1 relative to the reactant complex R•AA was calculated to be 26.9 kcal·mol^−1^ in water. This value is higher than that of TS-2 (see below) by 9 kcal·mol^−1^. Therefore, the first step is predicted to be the rate-determining step. Moreover, the value of 26.9 kcal·mol^−1^ is very close to the activation barrier recently calculated for a three-water-catalyzed succinimide formation from Asp (26.7 kcal·mol^−1^) [24] and is plausible for a nonenzymatic reaction, which occurs slowly at room temperature or physiological temperature.

In a vacuum, the complex I•AA-1 is more stable than the separated state (I + AA) by about 5 kcal·mol^−1^. However, it becomes less stable than the separated state by 1.4 kcal·mol^−1^ when the hydration effect is taken into account. Moreover, there exists another intermediate complex (I•AA-2, Figure 7), which is much more stable than I•AA-1, both in a vacuum and in water. In water, I•AA-2 is more stable than I•AA-1 by 6.4 kcal·mol^−1^ and higher in energy than R•AA by 8.7 kcal·mol^−1^. In I•AA-2, the AA molecule forms two hydrogen bonds with the *gem*-diol group of I (Figure 7). The C=O oxygen of AA forms a hydrogen bond to one of the OH groups in the *gem*-diol moiety, and the OH hydrogen of AA forms a hydrogen bond to the oxygen of the other OH group in the *gem*-diol moiety. I•AA-1 and I•AA-2 have similar main-chain conformations, but χ_1_ is larger in I•AA-2 by 29° than in I•AA-1.

From I•AA-2, dehydration occurs via the transition state TS-2 (Figure 8) to give the product complex P•AA•W (Figure 9). In this step, one of the C−O bonds in the *gem*-diol moiety is cleaved (see Figure 8). In TS-2, the breaking C−O bond is elongated to 1.837 Å from 1.463 Å in I•AA-2. Concomitantly with this bond cleavage, a double proton transfer occurs mediated by the AA molecule. The OH hydrogen of AA moves toward the departing oxygen, leading to the formation of a water molecule. On the other hand, the hydrogen attached to the other oxygen of the *gem*-diol moiety moves toward the C=O oxygen of AA. In this process, the AA molecule again acts as both proton donor and acceptor. It should be noted that the local activation barrier of the second step is as low as 9.2 kcal·mol^−1^. The relative energy of TS-2 with respect to R•AA is 17.9 kcal·mol^−1^, which is lower than that of TS-1 by 9 kcal·mol^−1^. The resultant P•AA•W is a complex formed by P (Figure 3b), AA and W and is more stable than the separated state (P + AA + W) by 15.1 and 5.0 kcal·mol^−1^ in a vacuum and in water, respectively. The changes in φ, ψ and χ_1_ continuously from I•AA-2 to P•AA•W are very small. The energy of P•AA•W is lower than that of I•AA-2 by 14.8 kcal·mol^−1^.

When the energies of the initial separated state (R + AA) and the final separated state (P + AA + W) are compared, the latter was calculated to be more stable by 5.1 kcal·mol^−1^. Considering that the experimental hydration free energy of water is −6.3 kcal·mol^−1^ [58], while it is calculated to be −9.1 kcal·mol^−1^ at the present level of calculation, it may be said that the reactant and product states are very close to each other in energy. This is consistent with the fact that the Asp and Asu forms coexist in aqueous solution depending on the conditions [18,25,38,54].

The double proton transfers in the first and second steps, especially the latter, may remind one of the degenerate, synchronous double proton transfers occurring in the cyclic dimers of carboxylic acids, including the AA dimer [59,60,61]. The best theoretical estimate of the barrier height for the double proton transfer in the formic acid dimer is 8.94 kcal·mol^−1^ [59]. This value can be compared with the local activation barrier for the second step of the present reaction (8.4 and 9.2 kcal·mol^−1^ in a vacuum and in water, respectively). It should also be noted that carboxylic acids participate in many excited-state double-proton transfer (ESDPT) processes [62,63,64,65].

From the viewpoint of mechanistic organic chemistry, both the first and second steps can be viewed as a bond reorganization process occurring in a cyclic hydrogen-bonded complex. The first step (cyclization), in particular, has been regarded as a nucleophilic attack by the amide nitrogen. Although this is more or less correct, it may be better viewed as a concerted bond reorganization process, which bears some resemblance to pericyclic reactions, considering that amide nitrogens are generally recognized as poor nucleophiles.

## 3. Computational Details

Figure 1 shows the model compound used in the present study, in which an Asp residue is capped with Ace and Nme groups on the *N*- and *C*-termini, respectively. This compound has previously been used in related computational studies by us [22,23,24] and Catak* et al.* [21,66]. The two-step reaction pathway was explored for a reactant complex formed between the model compound and a catalytic AA molecule.

All calculations were performed by using Spartan’14 [67]. As in our previous studies [22,23,24], energy-minimum and transition state geometries were located in a vacuum without any constraints by the density functional theory (DFT) with the B3LYP functional and the 6-31+G(d,p) basis set. Vibrational frequency calculations were performed for all of the optimized geometries to confirm them as energy minima (with no imaginary frequency) or transition states (with a single imaginary frequency) and to correct the relative energies for ZPE. Intrinsic reaction coordinate (IRC) calculations were performed from the transition states followed by full geometry optimizations to confirm that each transition state connects two energy minima, as shown in Figure 2. Furthermore, hydration effects have been included by single-point calculations at the same level of theory employing the SM8 continuum model [56,57].

## 4. Conclusions

We have computationally shown that acetic acid (AA) (CH_3_COOH, not CH_3_COO^−^) can catalyze succinimide formation from Asp residues. The reaction proceeds by two steps, cyclization and dehydration, and an AA molecule catalyzes both steps by acting as both proton donor and acceptor in double-proton transfers. The size and shape of the carboxyl group (–COOH) seem to be almost perfect to enable the double-proton transfer, both in the cyclization and dehydration steps. The rate-determining step was predicted to be the cyclization step (first step). Since protein drugs, especially monoclonal antibodies, are often formulated in acetate buffer, the AA-catalyzed reaction as revealed here can be a pharmaceutical concern. Carboxylic acids other than AA are also expected to catalyze succinimide formation from Asp residues. Very recently, deamidation of asparagine (Asn) residues (which also proceeds via the succinimide intermediate) has been shown to be catalyzed by a variety of carboxylic acids [68]. A similar mechanism to the presently proposed one may also operate in the Asn deamidation reactions. From the viewpoint of mechanistic organic chemistry, the “nucleophilic” attack by the amide nitrogen of the peptide backbone may be viewed as nominal. The cyclization step may better be viewed as a concerted bond reorganization process.
